# Recurrence Rates and Patterns after Radical Resection of Lung Carcinoids

**DOI:** 10.3390/cancers16172978

**Published:** 2024-08-27

**Authors:** Erika Askildsen, Patrick Soldath, Seppo W. Langer, Mikkel Andreassen, Ulrich Knigge, René Horsleben Petersen

**Affiliations:** 1 Department of Clinical Medicine, University of Copenhagen, 2200 Copenhagen, Denmark; patrick.soldath@regionh.dk (P.S.); seppo.langer@regionh.dk (S.W.L.); rene.petersen@rh.regionh.dk (R.H.P.); 2 Department of Cardiothoracic Surgery, Copenhagen University Hospital—Rigshospitalet, 2100 Copenhagen, Denmark; 3 Department of Oncology, Copenhagen University Hospital—Rigshospitalet, 2100 Copenhagen, Denmark; 4 Department of Endocrinology, Copenhagen University Hospital—Rigshospitalet, 2100 Copenhagen, Denmark; mikkel.andreassen.01@regionh.dk (M.A.); ulrich.peter.knigge@regionh.dk (U.K.); 5 Department of Gastrointestinal Surgery, Copenhagen University Hospital—Rigshospitalet, 2100 Copenhagen, Denmark

**Keywords:** lung neuroendocrine neoplasms, atypical carcinoid, typical carcinoid, nodal involvement, resection margin

## Abstract

**Simple Summary:**

Lung carcinoids are known to be less malignant than other types of lung cancers. However, atypical lung carcinoids are more prone to recur after radical surgery than typical lung carcinoids. The aim of our retrospective study was to assess the rate of recurrence of low-stage atypical and typical carcinoids when accounting for competing events. We confirmed that atypical carcinoids recurred more often than typical carcinoids within 5 and 10 years after radical surgery. Atypical carcinoids at a low stage are more prone to recur after radical surgery, suggesting that all patients operated for atypical carcinoid should undergo close follow-up care after surgery.

**Abstract:**

Atypical lung carcinoid (AC) is widely accepted to recur more often after radical resection than typical lung carcinoid (TC). However, their recurrence rates have never been compared in a multi-state competing risks model. We retrospectively reviewed files from patients with AC and TC who had been radically resected at our European Neuroendocrine Tumor Society Center of Excellence between 2009 and 2020. We estimated the recurrence rates between the AC and TC patients counting unrelated death as a competing event using Aalen–Johansen estimates and compared them using a multi-state Cox model. Finally, we analyzed all AC and TC recurrences as to resection type, pathological stage, resection margin, recurrence site, and time to recurrence. The study included 217 patients, of whom 194 had TC and 23 had AC. The median follow-up was 9.4 years. The AC patients experienced recurrence at a higher rate (hazard ratio [HR] 16.0, 95% confidence interval [CI] 5.3–47.9, *p* < 0.001). Correspondingly, the 5- and 10-year recurrence rates were 18% and 32% for AC and merely 1.0% and 2.4% for TC. In patients without nodal involvement, AC recurred at a considerably higher rate (HR 41.2, 95% CI 8.7–194.8, *p* < 0.001) than TC. In both AC and TC, most recurrences were distant and occurred in patients with a resection margin less than 2 cm. We conclude that AC recurs more often than TC, even in patients without nodal involvement at surgery, suggesting that all AC patients regardless of their pathological stage should undergo close follow-up care after surgery.

## 1. Introduction

Typical (TC) and atypical lung carcinoid (AC) are rare neuroendocrine neoplasms of the lung. While both carcinoids are classified as well-differentiated neoplasms, TC is graded as a low malignancy, whereas AC is graded as an intermediate malignancy [1]. In clinical practice, AC is also widely accepted as being more prone to recur after radical resection than TC [2]. However, the recurrence rates have never been compared in a multi-state competing risks model. Considering their relatively low malignancies, using a proper competing risks method is crucial to estimate the true recurrence rates. Knowing the true recurrence rates will aid lung cancer physicians in composing carcinoid-specific follow-up care programs for the benefit of patients and health services. 

In this study, we estimate and compare the recurrence rates of TC and AC using a multi-state competing risks model. In addition, we analyze all TC and AC recurrences as to the patients’ resection type, pathological stage, resection margin, recurrence site and way of diagnosis, and time to recurrence.

## 2. Materials and Methods 

We reviewed a prospectively maintained database of patients with TC and AC who had been radically resected at our European Neuroendocrine Tumor Society Center of Excellence between 2009 and 2020. We extracted the patients’ demographical, clinical, and pathological data from our database and their recurrence and survival status as well as the cause of death from medical records and the Danish Social Security Death Index. Recurrence and survival status were censored on 17 May 2024. The study was approved by the Danish Patient Safety Authority (ref. no. 31-1521-297), the Capital Region of Denmark (ref. no. 2007-58-0015), and the Institutional Review Board. Patient informed consent was not required.

All patients were diagnosed, treated, and followed according to the guidelines of the European Society for Medical Oncology [3]. Whenever possible, video-assisted thoracoscopic surgery (VATS) was used [4], while thoracotomy was reserved for patients with large tumors (>7–8 cm), central tumors that needed a sleeve resection, or tumors that involved the chest wall. Lobectomy and extended variants were the standard operations, while segmentectomy was an option in small peripheral tumors. Wedge resections were only used in patients with a compromised lung function who would not tolerate a larger resection. All anatomical resections were followed by a systematic nodal dissection [5], whereas wedge resections were followed by nodal sampling at the surgeon’s discretion. Tumors were classified as TC or AC and lymph nodes as benign or malignant according to the World Health Organization classification on thoracic tumors [1]. Resection margin was classified as above or below 2 cm from the border of the tumor to the closest resection line. Tumors were staged according to the 8th edition of the American Joint Committee on Cancer staging manual [6]. They were defined as central if they were visible on a bronchoscopy.

Patients were intentionally followed for 10 years after surgery. In some cases, the follow-up was modified at the clinician’s discretion. Computed tomography (CT) scans of the chest and upper abdomen were performed at 3, 6, and 12 months after surgery and thereafter every 12 months. In patients with central tumors, a bronchoscopy was also aspired to be performed at the same intervals until 5 years from surgery. Patients who were suspected of recurrence on either a CT scan or bronchoscopy were evaluated with somatostatin receptor (SSTR)-positron emission tomography (PET)/CT scan and, if technically possible, a subsequent biopsy or resection of the lesion. Recurrence was diagnosed from a positive pathological specimen or a positive lesion on an SSTR-PET/CT scan. Local recurrence was specified as lesions at the resection line or in the parenchyma of the same lobe in patients who had a sublobar resection. Regional recurrence was specified as dissemination to the hilar (N1) or mediastinal nodes (N2) and distant recurrence as lesions involving all other locations, including pleural and pericardial effusions. 

### Statistical Analysis

For the recurrence analysis, we used a multi-state competing risks model that counted death without recurrence as a competing event. We estimated the recurrence rates of TC and AC using the Aalen–Johansen estimates at 5 and 10 years from surgery and compared the rate of recurrence between TC and AC using a multi-state Cox model. Because relatively more AC patients had nodal involvement at surgery, we repeated the analysis on the subgroups of TC and AC patients without nodal involvement at surgery. We estimated the median follow-up using the reverse Kaplan–Meier method.

Summary statistics are presented as median and interquartile range (IQR). Continuous variables were compared using the Wilcoxon rank sum test and categorical variables using the Pearson’s Chi-squared test or Fisher’s exact test as appropriate. All *p*-values were two-sided and those less than or equal to 0.05 were considered statistically significant. All data were analyzed using R statistical computing software (version 4.4.1, R Core Team 2024). The full reproducible code is available in Supplementary Materials.

## 3. Results

The study included 217 patients, of whom 194 had TC and 23 had AC. The patients’ demographical, clinical, and pathological data are summarized in Table 1. 

Notably, the AC patients were older and more often presented with pathological nodal involvement. The patients’ pathological tumor and nodal categories matched well with their clinical ones. The clinical and pathological tumor and nodal categories are listed in Table 2. 

The median follow-up was 9.4 years (6.7–12.6 years). During follow-up, seven patients with AC (30.4%) and five with TC (2.6%) experienced recurrence. Most of the recurrences were distant (especially to the liver) and all but one occurred in patients with a resection margin less than 2 cm. Furthermore, most of the recurrences were diagnosed in asymptomatic patients on scheduled follow-up scans. The patients’ recurrence patterns as well as their pathological and follow-up data are listed in Table 3.

**Figure 1:**
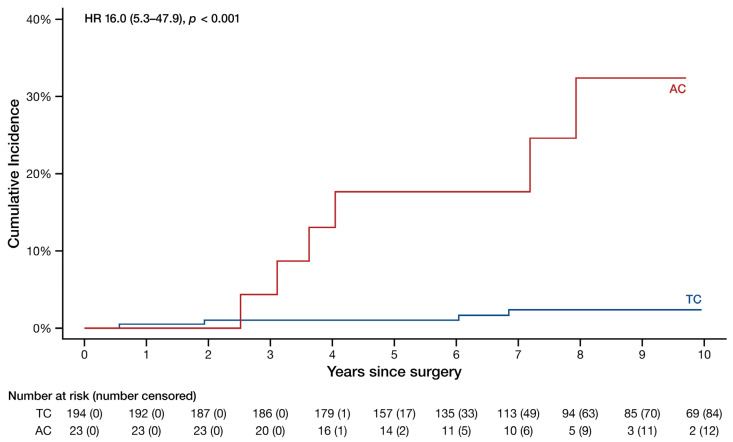
Cumulative incidence curves of the recurrence probability of all typical and atypical carcinoid patients. HR: hazard ratio (95% confidence interval). Note that the y-axis spans from 0% to 40%.

The AC patients experienced recurrence at a higher rate (hazard ratio [HR] 16.0, 95% confidence interval [CI] 5.3–47.9, *p* < 0.001) visualized in Figure 1. Correspondingly, the 5- and 10-year recurrence rates were 18% and 32% for AC and merely 1.0% and 2.4% for TC. The median time to recurrence was 4.1 years (3.4–7.6 years) for AC and 6.0 years (1.9–6.8 years) for TC.

**Figure 2:**
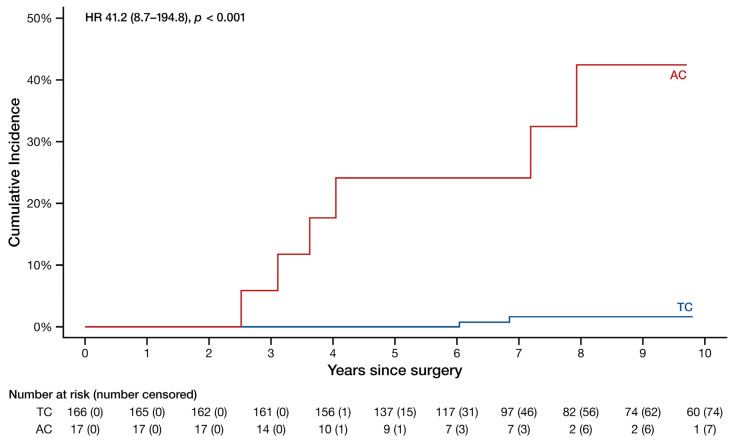
Cumulative incidence curves of the recurrence probability of typical and atypical carcinoid patients without nodal involvement. HR: hazard ratio (95% confidence interval). Note that the y-axis spans from 0% to 50%.

In patients without nodal involvement at surgery, AC recurred at a considerably higher rate (HR 41.2, 95% CI 8.7–194.8, *p* < 0.001) than TC as visualized in Figure 2. The 5- and 10-year recurrence rates in these patients were 24% and 42% for AC and 0.0% and 1.6% for TC, while the median time to recurrence was 3.8 years (3.2–6.4 years) for AC and 6.9 years (6.4–9.4 years) for TC.

A total of 76 patients had central tumors, of whom 44 underwent the 5-year bronchoscopy follow-up program. Of these, 34 completed the program. No patients were diagnosed with a recurrence on bronchoscopy; however, 14 patients were found to have complications requiring bronchoscopic intervention at one or more examinations. Of these, 13 patients had undergone a sleeve resection. The complications included 1 incidence of fistula requiring stent treatment, 9 incidences of granuloma requiring laser resection, 14 incidences of loose sutures requiring removal, and 3 incidences of stenosis requiring balloon dilatation. About half of the complications occurred in the first 2 years and the other half in the last 3 years. All 44 patients and their bronchoscopic follow-up examinations including complications are visualized in Supplementary Figure S1.

## 4. Discussion 

To the best of our knowledge, this is the first study to estimate and compare recurrence rates of TC and AC using multi-state competing risks models. Our cohort of TC and AC patients is one of the largest originating from a single center and one of the longest surveilled. The main findings are that AC recurs at a considerably higher rate than TC, especially in patients without nodal involvement. Furthermore, both types usually recur distantly instead of locally or regionally and almost exclusively in patients with a resection margin less than 2 cm. While several older and newer studies have investigated survival rates in TC and AC [7,8,9,10,11,12,13,14,15,16,17], very few have investigated the recurrence rates and patterns and almost all of them using statistical methods that do not account for competing risks [15,16,17,18]. Failure to account for competing risks can lead to overestimates of the cumulative incidences. This bias is more pronounced in diseases with a low incidence of events, such as AC and especially TC.

The only other study that analyzed recurrence in TC and AC after radical resection using a competing risk model is the single-center case-series by Filosso and colleagues from 2013 [19]. The authors primarily investigated Kaplan–Meier overall survival rates of 83 TC and 43 AC patients, but they also conducted a subanalysis on patients who developed distant metastasis using a Fine–Gray regression model. Their results showed that AC and nodal involvement were independent prognostic factors for developing distant metastasis. While they did not investigate whether the same was true for the development of local or regional recurrence (presumably due to insufficient events), their results are rather comparable to our findings. As stated, we found markedly higher recurrence rates at 5 and 10 years in AC patients than in TC patients, but we also observed higher recurrence rates in TC patients with nodal involvement compared with those without nodal involvement. Unfortunately, we were not able to analyze the latter in a Cox model due to insufficient events. On the other hand, we did not observe higher recurrence rates in the AC patients with a nodal involvement than in those without a nodal involvement, which might be explained by the small cohort of AC patients. Filosso and colleagues also investigated the recurrence patterns and found similar distributions of distant, regional, and local recurrences to our findings. However, their distribution of distant recurrence was somewhat different with most presenting in the brain, the second most in the liver, and the third most in the bone. 

As stated, many studies have analyzed survival rates of TC and AC after radical resection. Survival rates are easier to investigate as the survival status may be simply extracted from a central social security death index, whereas the recurrence status can only be obtained by reviewing the patients’ medical records and scan reports. While survival rates are perfectly usable in deadly diseases with a relatively short survival time, they are not so useful in the opposite case as patients would need to be followed for a very long time to observe their event. Such a long timeframe is often not feasible even in retrospective studies. As recurrence precedes death, using it instead of survival will typically lead to a higher number of events within the same follow-up period allowing for more accurate risk estimates. This is particularly true in low-malignant cancers, in which patients may live for a long time after recurrence. Two recent registry-based studies investigated cancer-specific survival rates in TC and AC using death as a competing risk [20,21]. The study by Yoon and colleagues analyzed 4254 TC and 391 AC patients, of whom 86% had undergone a surgical resection. With a median follow-up time of 4.5 years, they analyzed 10-year survival rates and found somewhat lower cumulative incidences (converted) in both TC and AC patients overall compared with our findings. The study by Wang and colleagues investigated 2223 TC and 273 AC patients, of whom 84% had undergone a surgical resection. With a median follow-up time of 6.3 years, they found a higher cumulative incidence in TC patients overall (around 10%) compared with our 2.4% but similar cumulative incidence in AC patients overall (around 35%) compared with our 32%. The findings of these two studies compared with our findings confirm that survival rates may underestimate the malignancy of low-malignant cancers compared with recurrence rates.

No patients with AC had Ki-67 over 20%, making the AC patients homogeneous and without potential confounders in the form of supracarcinoids.

In our study, none of the 44 patients who had central tumors and went through the 5-year bronchoscopy follow-up program were bronchoscopically diagnosed with a recurrence. A recent study from another Danish center investigated 153 TC and 29 AC patients as well as 26 patients with large cell neuroendocrine carcinoma, of whom 118 underwent yearly bronchoscopic follow-up after a surgical resection of central tumors [22]. The authors found that only 2 out of their 22 total recurrences were detected on bronchoscopic examinations; however, those 2 recurrences were also detected on the follow-up scans. The authors did not report on the histology of the recurrences but considering our and their findings, bronchoscopic follow-up in central TC and AC may be redundant. 

While we did not detect any recurrence on our bronchoscopic follow-up examinations, we did find 14 patients who had treatment-requiring complications at one or more examinations. As all but one of these cases were patients who had undergone a sleeve resection and half of the complications occurred in the last 3 years of the follow-up program, there is still a clear indication for following patients who have undergone a sleeve resection for a minimum of 5 years after surgery.

Our study has some limitations. First, it is limited by its retrospective design, which inherently introduces a selection bias. Nevertheless, our cohort was quite uniform with more than 90% of patients receiving an anatomical resection and most of these being lobectomies. Second, although, our cohort is one of the largest described in the literature, it only included 23 ACs, making our comparisons between TC and AC susceptible to some inaccuracy. A strength of our cohort, however, is that all the patients were operated at a single center within a relatively short timeframe using the very same approaches across all treatment aspects.

In conclusion, this study supports existing evidence that AC recurs at a considerably higher rate than TC, especially in patients without nodal involvement, suggesting that all AC patients regardless of their pathological stage demand a proper follow-up care after surgery, which should entail yearly ambulatory CT scans for at least 10 years. As to their patterns of recurrence, both TC and AC usually recur distantly instead of locally or regionally and almost exclusively in patients with a resection margin of less than 2 cm.

## Figures and Tables

**Table 1:** Demographical, clinical, and pathological characteristics of the typical and atypical carcinoid patients. AC: atypical carcinoid, DLCO: diffusion capacity for carbon monoxide, FEV1: forced expiratory volume in the first second in percent of predicted value, TC: typical carcinoid, VATS: video-assisted thoracoscopic surgery.

Characteristic	Overall N = 217 ^1^	TC N = 194 ^1^	AC N = 23 ^1^	*p*-Value ^2^
Age	64 (53–71)	63 (52–70)	70 (64–73)	0.016
Sex				>0.99
Female	151 (70)	135 (70)	16 (70)	
Male	66 (30)	59 (30)	7 (30)	
Smoking history				0.96
Current	34 (16)	30 (15)	4 (17)	
Former	99 (46)	89 (46)	10 (43)	
Never	84 (39)	75 (39)	9 (39)	
FEV1	88 (76–101)	89 (76–101)	88 (75–107)	0.91
Unknown	4	4	0	
DLCO	80 (66–89)	79 (65–89)	83 (69–88)	0.46
Unknown	82	77	5	
Tumor location				0.63
Central	76 (35)	69 (36)	7 (30)	
Peripheral	141 (65)	125 (64)	16 (70)	
Surgical approach				0.35
Thoracotomy	55 (25)	51 (26)	4 (17)	
VATS	162 (75)	143 (74)	19 (83)	
Surgical resection				0.92
Bilobectomy	8 (3.7)	8 (4.1)	0 (0)	
Bronchial sleeve	5 (2.3)	5 (2.6)	0 (0)	
Lobectomy	150 (69)	132 (68)	18 (78)	
Pneumonectomy	1 (0.5)	1 (0.5)	0 (0)	
Segmentectomy	6 (2.8)	5 (2.6)	1 (4.3)	
Sleeve lobectomy	27 (12)	25 (13)	2 (8.7)	
Wedge	20 (9.2)	18 (9.3)	2 (8.7)	
Pathological T-category				0.43
1a	30 (14)	29 (15)	1 (4.3)	
1b	90 (41)	76 (39)	14 (61)	
1c	35 (16)	33 (17)	2 (8.7)	
2a	42 (19)	38 (20)	4 (17)	
2b	6 (2.8)	6 (3.1)	0 (0)	
3	12 (5.5)	10 (5.2)	2 (8.7)	
4	2 (0.9)	2 (1.0)	0 (0)	
Pathological N-category				0.03
0	183 (84)	166 (86)	17 (74)	
1	10 (4.6)	7 (3.6)	3 (13)	
2	11 (5.1)	8 (4.1)	3 (13)	
Unknown	13 (6.0)	13 (6.7)	0 (0)	
Resection margin				0.17
Less than 2 cm	139 (66)	121 (64)	18 (78)	
More than 2 cm	73 (34)	68 (36)	5 (22)	
Unknown	5	5	0	
Ki-67	4.0 (2.0–6.0)	4.0 (2.0–5.0)	14.0 (10.0–20.0)	<0.001
Unknown	6	6	0	

^1^ Median (Q1–Q3); n (%), ^2^ Wilcoxon rank sum test; Pearson’s Chi-squared test; Fisher’s exact test.

**Table 2:** Clinical and pathological tumor and nodal categories of the typical and atypical carcinoid patients. AC: atypical carcinoid, N-category: nodal category, TC: typical carcinoid, T-category: tumor category.

	TC		AC	
Category	Clinical, N = 194 ^1^	Pathological, N = 194 ^1^	Clinical, N = 23 ^1^	Pathological, N = 23 ^1^
T-category				
T1	115 (59)	138 (71)	17 (74)	17 (74)
T2	53 (27)	44 (23)	5 (22)	4 (17)
T3	13 (6.7)	10 (5.2)	1 (4.3)	2 (8.7)
T4	9 (4.6)	2 (1.0)		
Unknown	4 (2.1)			
N-category				
N0	166 (86)	166 (86)	20 (87)	17 (74)
N1	12 (6.2)	7 (3.6)	3 (13)	3 (13)
N2	11 (5.7)	8 (4.1)		3 (13)
Unknown	5 (2.6)	13 (6.7)		

^1^ N (%).

**Table 3:** Recurrence patterns, pathological and follow-up characteristics of the typical and atypical carcinoid patients. AC: atypical carcinoid, CRT: chemoradiotherapy, N-category: nodal category, PRRT: peptide receptor radionuclide therapy, RFA: radiofrequency ablation, SSA: somatostatin analog, TC: typical carcinoid, T-category: tumor category, WBRT: whole brain radiation therapy.

Subtype	Resection Type	Tumor Location	Pathological T-Category	Pathological N-Category	Resection Margin	Ki-67	Recurrence Type	Recurrence Site	Recurrence Diagnosis	Histological Confirmation	Years to Recurrence	Recurrence Treatment	Status	Cause of Death
TC	Lobectomy	Peripheral	1b	N0	Below 2 cm	3	Distant	Contralateral lung	Coughing	Yes	6.0	Segmentectomy	Alive	—
TC	Sleeve lobectomy	Central	3	N0	Below 2 cm	5	Local, regional, and distant	Ipsilateral lung, mediastinum, and bones	Dyspnea	No	11.9	None	Deceased	Disease related
TC	Bilobectomy	Central	1c	N0	Below 2 cm	8	Distant	Liver	Scheduled scan	Yes	6.8	PRRT and SSA	Alive	—
TC	Wedge	Peripheral	1a	Unknown	Below 2 cm	3	Local	Ipsilateral lung	Scheduled scan	No	1.9	None	Alive	—
TC	Wedge	Peripheral	1a	Unknown	Below 2 cm	5	Local	Ipsilateral lung	Scheduled scan	Yes	0.6	Wedge resection and SSA	Alive	—
AC	Lobectomy	Central	1b	N0	Below 2 cm	13	Distant	Liver	Scheduled scan	Yes	7.9	Microwave ablation	Alive	—
AC	Lobectomy	Central	3	N0	Below 2 cm	13	Distant	Ileum	Ileus	Yes	7.2	Bowel resection	Alive	—
AC	Lobectomy	Peripheral	1a	N0	Below 2 cm	10	Distant	Liver	Scheduled scan	No	3.6	SSA	Deceased	Disease related
AC	Lobectomy	Peripheral	1b	N0	Below 2 cm	15	Local and distant	Both lungs and right adrenal gland	Scheduled scan	Yes	2.5	SSA	Deceased	Disease related
AC	Lobectomy	Peripheral	1b	N0	Above 2 cm	20	Local, regional, and distant	Mediastinum, liver, and bones	Scheduled scan	No	4.0	PRRT and SSA	Deceased	Disease related
AC	Lobectomy	Peripheral	2a	N0	Below 2 cm	16	Distant	Pancreas and brain	Headache	Yes	3.1	Brain resection and WBRT	Deceased	Disease related
AC	Lobectomy	Peripheral	2a	N1	Below 2 cm	10	Distant	Liver	Scheduled scan	Yes	14.2	SSA	Deceased	Disease related

## Data Availability

The original contributions presented in the study are included in the article/Supplementary Material; further inquiries can be directed to the corresponding author/s.

## Supplementary Materials

The following supporting information can be downloaded at: https://www.mdpi.com/article/10.3390/cancers16172978/s1, Figure S1: Swimmer plot of the typical and atypical carcinoid patients with central tumors who underwent the 5-year bronchoscopy follow-up program.
